# Pregnancy prevalence and outcomes after optic neuritis in South Korea

**DOI:** 10.1038/s41598-023-38851-x

**Published:** 2023-07-27

**Authors:** Daye Diana Choi, Kyung-Ah Kim, Kyung-Ah Park, Sei Yeul Oh

**Affiliations:** 1 Department of Ophthalmology, Kim’s Eye Hospital, Seoul, Korea; 2 Statistics and Data Center, Samsung Medical Center, Seoul, Korea; 3 Department of Ophthalmology, Samsung Medical Center, Sungkyunkwan University School of Medicine, 81 Irwon-ro, Gangnam-gu, Seoul, 06351 Korea

**Keywords:** Diseases, Medical research, Risk factors

## Abstract

To compare pregnancy rates and complications in women with and without a history of optic neuritis (ON). A nationwide, population-based, retrospective study using data from the Korean National Health Claims from January 2011, to December 2017 was done. ON cohort (ON group) consisting of women aged 18 to 50 with a history of ON and 1:3 age-matched controls (control group) were compared for pregnancy and delivery rates using logistic regression after adjusting for possible confounders. Pregnancy-ON cohort (pregnancy-ON group), women aged 18 to 55 with a history of ON and pregnancy, and 1:3 age at pregnancy matched controls (pregnancy-control group) were analyzed for pregnancy complications using logistic regression after adjusting for covariates. ON group (*n* = 2516) showed decreased odds ratio (OR) for pregnancy [Adjusted OR2: 0.716, 95% confidence interval (CI): 0.626–0.820] and delivery (adjusted OR2: 0.647, 95% CI: 0.554–0.756) compared to controls (*n* = 7548). Pregnancy-ON group (*n* = 550) showed increased risk of delayed fetal growth (adjusted OR2: 9.867, 95% CI: 1.224–79.564), pre-eclampsia (adjusted OR2: 8.327, 95% CI: 2.911–23.819), preterm delivery (adjusted OR2: 3.914, 95% CI: 2.667–5.742), pregnancy and postpartum infection (adjusted OR1: 1.671, 95% CI: 1.296–2.154), diabetes in pregnancy (adjusted OR2: 1.365, 95% CI: 1.062–1.754) compared to pregnancy-control group (*n* = 1650). Our population-based cohort study suggests that history of ON is associated with decreased pregnancy and delivery rates. It may be a risk factor for various pregnancy complications.

## Introduction

Optic neuritis (ON) is an acute inflammatory optic nerve disorder that can cause significant visual loss. It is one of the most common optic neuropathies in adults along with glaucoma^1^. ON is often associated with various underlying diseases such as multiple sclerosis (MS), neuromyelits optica spectrum disorders (NMOSD)^2^, myelin oligodendrocyte glycoprotein antibody-associated disease (MOGAD) which is relatively recently recognized^3^, and rheumatic diseases. The development of demyelinating ON, the most common type of ON, is believed to be immune-mediated like its commonly accompanying disorders such as MS, NMOSD, MOGAD, and rheumatic diseases. It is well known that the incidence of ON as well as MS, NMOSD, and rheumatic diseases is higher in females than in males^4–8^. The higher immune reactivity in females has been suggested to predispose a female preponderance of diseases with an autoimmune nature^9^. These diseases including ON frequently appear at childbearing age. Previous studies revealed that most autoimmune disorders including MS, NMOSD, and rheumatic disorders pose a risk for almost all aspects of reproduction, ranging from fertility to pregnancy itself^10–14^. However, little is known about pregnancy outcomes in patients with ON without any other known systemic disease. Thus, this study aimed to compare pregnancy, abortion, and delivery rates in women with ON history to healthy controls without ON history using Korean Health Insurance Review and Assessment (HIRA) data. The HIRA of South Korea holds all health care utilization information of the entire population of 50 million people, owing to the mandatory universal health insurance system. A nationwide registry for rare and intractable diseases (the Rare Diseases Registry; RDR) was also established in 2009 by the Korean National Health Insurance in cooperation with the Ministry of Health and Welfare. Patients with MS or NMOSD, known to be major neurologic disorders frequently associated with ON, are registered in the RDR. In this study, we also examined and compared the risk of pregnancy complications in women with ON history or without ON history (pregnancy-control group).

## Methods

### Data source

We accessed health claims data recorded by the HIRA service of South Korea from January 1, 2011, to December 31, 2017. This database holds all inpatient and outpatient health care utilization information using codes from the *Korean Standard Classification of Diseases*, 7th Revision based on the International Classification of Diseases, 10th Revision. Patients in the HIRA database can be identified by their unique Korean Resident Registration Number assigned to each South Korean resident at birth. We had access to all medical claims of each enrollee. The HIRA Deliberative Committee approved the conditional use of the HIRA database for access to data collected from 2009 through 2017 for the purpose of this study. Anyone can assess the same raw data used in this paper. However, HIRA’s audited approval is mandatory. This study was performed in accordance with the principles of the Declaration of Helsinki. It was approved by Samsung Medical Center Institutional Review Board(IRB no. SMC 2019-01-025). Since the health insurance data we used were completely unidentifiable, the requirement of informed consent was waived by the Samsung Medical Center Institutional Review Board.

### Definitions of pregnancy, delivery, and abortion

Pregnancy was defined as if a subject had a history of more than one claim for pregnancy, abortion, delivery, pregnancy complications, or delivery complications. Abortion was defined to have a claim code for abortion. Delivery was defined if a subject had a claim code for delivery and more than two days of hospitalization or a claim for pregnancy and delivery complication codes. Diagnostic codes for each definition are shown in Supplemental Table 1.

### Optic neuritis cohort and controls for rates of pregnancy, delivery, and abortion

If the cohort consisted of women aged 18 to 55 years, ON and control cohorts could be matched 1:2 by age. To yield higher precision, 1:3 matching was chosen for this study. The ON cohort consisted of women aged 18 to 50 years who had claim code for ON (H46) twice from 2009 to 2012. Any subject who had a history of medical visit with diagnostic code for MS, NMOSD, acute transverse myelitis (ATM), or rheumatic diseases before the onset of the first visit with ON claim code was excluded from the ON cohort.

For the control group, among women who never had ON claim code, 1:3 exact age-matched subjects were enrolled. Age at ON diagnosis in the ON group and age at the first visit in the control group were used for matching. ON and control groups were followed up for five years. In the ON cohort, claims for pregnancy, abortion, and delivery only after ON diagnosis were included for each rate calculation.

Crude and adjusted odds ratio (ORs) of pregnancy, abortion, and delivery for the ON group were calculated and compared to the control group. Any comorbid disease with a *p*-value less than 0.2 was adjusted for OR2. Definitions and lists of comorbid diseases are shown in Supplemental Table 2.

### Pregnancy-optic neuritis cohort (pregnancy-ON group) and pregnancy-control group

The pregnancy-ON group consisted of women aged 18 to 55 years who had a claim code for ON (H46) more than two times and a history of pregnancy from 2009 to 2017. Any subject who had a history of medical visits with diagnostic codes for MS, NMOSD, ATM, or rheumatic diseases before the onset of the first visit with the ON claim code was excluded from the ON cohort.

For the pregnancy-control group, 1:3 exact age-matched subjects were enrolled among women who never had ON claim code but had a history of pregnancy. Age at pregnancy was used for matching for the two groups.

The OR of pregnancy complications for the pregnancy-ON group compared to the pregnancy-control group was calculated. Adjusted OR1 was adjusted for age at pregnancy, and adjusted OR2 was further adjusted for any comorbidities associated with the outcome of interest. Any comorbid disease with a *p*-value less than 0.2 was adjusted for OR2.

### Definition of pregnancy complications

Pregnancy complications analyzed in our study were pre-eclampsia, eclampsia, gestational hypertension, diabetes mellitus in pregnancy, placenta previa, premature separation of the placenta, preterm delivery, pregnancy or postpartum infection, delayed fetal growth, and Cesarean section (C-sec). Claim codes for each disease are shown in Supplemental Table 1.

### Statistical analysis

SAS Enterprise version 6.1 software program (SS Institute, Cary, NC, USA) was used for all statistical analyses. For comparisons, the Chi-squared test and Fisher’s exact test were used. Logistic analysis was used to calculate the odds ratio of pregnancy, delivery, and abortion rate, as well as pregnancy complications. A *p*-value of less than 0.05 indicated statistical significance.

## Result

In the HIRA database, there were 33,019 subjects aged 0–90 years who had two times or more claim codes for ON (H46) from 2009 to 2012. Among them, a total of 2516 women aged 18–50 years were categorized as the ON cohort group in this study. There were 363,622 women aged 18–50 years without a history of ON claim code. Among them, 7548 were selected by 1:3 exact age matching with the ON group and categorized as the control group.

### Optic neuritis cohort vs. controls for rates of pregnancy, delivery, and abortion

The mean age at pregnancy was older in the ON group (31.61 years, standard deviation [SD]: 6.48 years) than in the control group (30.30 years, SD: 9.48 years) (Table 1). Pregnancy, delivery, and vaginal delivery rate were significantly higher in the control group than in the ON group, while abortion and C-sec rates were not significantly different between the two groups. Many comorbid autoimmune diseases including MS, NMOSD, and ATM were more frequent in the ON group than in the control group. On the contrary, rates of subjects with hypertension, dyslipidemia, thyroid disease, and endometriosis were higher in the control group than in the ON group (Table 1).

**Table 1:** Clinical characteristics of the Korean female optic neuritis group and control group matched at 1:3.

	ON group (N = 2516)		Control group (N = 7548)		*P*-value	SMD
Age at baseline^a^ (mean ± SD)	36.01 ± 9.48		36.01 ± 9.48		1	0
Age of pregnancy (mean ± SD)	31.61 ± 5.53		30.30 ± 5.33		** < 0.0001**	0.241
Pregnancy (N, %)	354	14.07	1356	17.97	** < 0.0001**	0.106
Delivery (N, %)	242	68.36	1024	75.52	** < 0.0001**	0.124
Caesarean section	101	41.74	373	36.43	0.0570	0.045
Vaginal delivery	127	52.48	613	59.86	** < 0.0001**	0.124
Others	14	5.79	38	3.71	0.7481	0.007
Abortion (N, %)	98	27.68	293	21.61	0.9762	0.001
Autoimmune diseases (N, %)						
Multiple sclerosis	63	2.5	0	0	** < 0.0001**	0.227
Neuromyelitis optica spectrum disorders	71	2.82	0	0	** < 0.0001**	0.241
Acute transverse myelitis	14	0.56	0	0	< 0.0001*	0.106
Systemic lupus erythematosus	8	0.32	3	0.04	0.0012*	0.066
Bechet or Sjorgen syndrome	16	0.64	4	0.05	** < 0.0001**	0.100
Sarcoidosis	6	0.24	1	0.01	0.0013*	0.064
Cronhn’s disease	1	0.04	1	0.01	0.4375*	0.016
Ulcerative colitis	3	0.12	4	0.05	0.3769*	0.023
Acute disseminated encephalomyelitis	21	0.83	1	0.01	** < 0.0001**	0.127
Systemic sclerosis	4	0.16	1	0.01	0.0156*	0.050
Autoimmune thyroiditis	33	1.31	0	0	** < 0.0001**	0.163
Acute rheumatic fever	2	0.08	3	0.04	0.6044*	0.016
Rheumatic arthritis	53	2.11	0	0	** < 0.0001**	0.207
Cancer (N, %)	98	3.9	276	3.66	0.5839	0.013
Diabetes mellitus (N, %)	134	5.33	428	5.67	0.5146	0.015
Hypertension (N, %)	137	5.45	687	9.1	** < 0.0001**	0.141
Dyslipidemia (N, %)	367	14.59	1354	17.94	**0.0001**	0.091
Alcohol addiction (N, %)	9	0.36	28	0.37	0.9242	0.002
Thyroid disease (N, %)	261	10.37	974	12.9	**0.0008**	0.079
Obesity (N, %)	4	0.16	20	0.26	0.3452	0.023
Ovarian dysfunction (N, %)	31	1.23	84	1.11	0.6260	0.011
Endometriosis (N, %)	41	1.63	174	2.31	**0.0424**	0.049

*P*-values in bold indicate statistically significant differences between the two groups.

*N* numbers, *ON* optic neuritis, *SMD* standardized mean difference.

*P-value by Fisher’s exact analysis, other were analyzed by Chi-squared test.

^a^The definition of age at baseline was the age at optic neuritis diagnosis for the optic neuritis group and the age of the first visit for the control group.

Supplemental Table 3 shows the odds ratio of comorbid diseases according to each pregnancy, delivery, and abortion in the ON group and the control group.

Regarding the odds ratio of pregnancy in the ON group, the crude odds ratio was 0.748 (95% CI: 0.659–0.849) compared to controls (Table 2). After adjusting for age at baseline, the OR1 was 0.717 (95% CI: 0.626–0.821). Adjusted OR2 with further adjustment for comorbid diseases associated with pregnancy rate was 0.716 (95% CI: 0.626–0.820).

**Table 2:** Odds ratios for pregnancy, delivery, abortion in the optic neuritis group compared to the control group.

	ON group, N (%)	Control, N (%)	Crude OR (95% CI)	Adjusted OR1^a^ (95% CI)	Adjusted OR2 (95% CI)
Pregnancy	354 (14.07)	1356 (17.97)	**0.748 (0.659, 0.849)**	**0.717 (0.626, 0.821)**	**0.716 (0.626, 0.820)**^b^
Delivery	242 (68.36)^c^	1024 (75.52)	**0.678 (0.585, 0.786)**	**0.650 (0.557, 0.759)**	**0.647 (0.554, 0.756)**^d^
Abortion	98 (27.68)^c^	293 (21.61)	1.004 (0.795, 1.267)	1.004 (0.793, 1.270)	1.001 (0.790, 1.268)^e^

Odds ratios shown in bold indicate numbers that are statistically significant based on the 95% confidence limit.

*N* numbers, *ON* optic neuritis, *OR* odds ratio, *CI* confidence interval.

^a^Adjusted for age at baseline.

^b^Adjusted for cancer, diabetes, hypertension, dyslipidemia, alcohol addiction, thyroid disease, and ovarian dysfunction.

^c^Percentage among pregnant subjects.

^d^Adjusted for Crohn’s disease, cancer, diabetes, hypertension, dyslipidemia, and ovarian dysfunction.

^e^Adjusted for hypertension, thyroid disease, and ovarian dysfunction.

Regarding the odds ratio of delivery in the ON group, the crude odds ratio was 0.678 (95% CI: 0.585–0.786). The OR1 of delivery in the ON group was 0.650 (95% CI: 0.557–0.759) after adjusting for age at baseline. The OR2 of delivery in the ON group was 0.647 (95% CI: 0.554–0.756), after further adjusting for comorbid diseases associated with the delivery rate.

Regarding the odds ratio of abortion in the ON group, the crude odds ratio was 1.004 (95% CI: 0.795–1.267). The adjusted OR1 was 1.004 (95% CI: 0.793–1.270) after adjusting for age at baseline. The adjusted OR2 was 1.001 (95% CI: 0.790–1.268) after additional adjustment for comorbid diseases associated with the abortion rate.

### Pregnancy-optic neuritis group vs. pregnancy-control group for rates of pregnancy complications

There were 75,642 subjects with two times or more claims of ON aged 0–90 years during 2009–2017. Among them, the number of women subjects aged 18–55 years was 8628. After excluding those without a history of pregnancy during the study period, 1747 subjects remained. We also excluded anyone who had a pregnancy claim before the ON claim. Finally, a total of 550 women who had a history of pregnancy after ON during 2009–2017 were included for the pregnancy-ON cohort. A total of 1,650 women 1:3 matched with age at pregnancy were selected for the pregnancy-control group. Clinical characteristics of the pregnancy-ON group and pregnancy-control group are displayed in Table 3. Supplemental Table 4 shows the odds ratio of comorbid diseases according to each pregnancy complication in the pregnancy-ON group and the pregnancy-control group. Rates of pre-eclampsia, diabetes in pregnancy, preterm delivery, pregnancy or postpartum infection, and C-sec were significantly higher in the pregnancy-ON group than in the pregnancy-control group. We also analyzed ATM, Wegener’s granulomatosis, systemic lupus erythematosus, sarcoidosis, Crohn’s disease, Ulcerative colitis, acute disseminated encephalomyelitis, acute rheumatic fever, obstetric death, and obstetric embolism. However, no one in either group had a history of such diseases. The risk of pregnancy complication was significantly higher in the pregnancy-ON group than in the pregnancy-control group for pre-eclampsia (adjusted OR1: 9.189, 95% CI: 2.951–28.619, adjusted OR2: 8.327, 95% CI: 2.911–23.819, Table 4), diabetes mellitus in pregnancy (adjusted OR1 1.359, 95% CI: 1.058–1.746, adjusted OR2: 1.365, 95% CI: 1.062–1.754), preterm delivery (adjusted OR1: 3.917, 95% CI: 2.671–5.744, adjusted OR2: 3.914, 95% CI: 2.667–5.742), and delay of fetal growth (adjusted OR2: 9.867, 95% CI: 1.224–79.564).

**Table 3:** Clinical characteristics of the pregnancy-optic neuritis group and the pregnancy-control group.

	Pregnancy-ON group (N = 550)		Pregnancy-control group (N = 1650)		*P*-value	SMD
Age of pregnancy (Mean ± SD)	31.34 ± 5.38		31.34 ± 5.38		1.0000	0
Multiple sclerosis (N, %)	1	0.18	0	0	0.2500*	0.060
Neuromyelitis optica (N, %)	2	0.36	0	0	0.0624*	0.085
Autoimmune dieases (N, %)						
Bechet or Sjorgen syndrome	0	0	1	0.06	1.0000*	0.035
Autoimmune thyroiditis	2	0.36	0	0	0.0624*	0.085
Rheumatic arthritis	1	0.18	0	0	0.2500*	0.060
Cancer (N, %)	1	0.18	4	0.24	1.0000*	0.013
Diabetes mellitus (N, %)	6	1.09	22	1.33	0.6605	0.022
Hypertension (N, %)	3	0.55	9	0.55	1.0000*	0.000
Dyslipidemia (N, %)	18	3.27	58	3.52	0.7875	0.013
Alcohol addiction (N, %)	0	0	2	0.12	1.0000*	0.049
Thyroid disease (N, %)	26	4.73	58	3.52	0.1989	0.061
Obesity (N, %)	0	0	4	0.24	0.5777*	0.070
Ovarian dysfunction (N, %)	10	1.82	26	1.58	0.6980	0.019
Entometriosis (N, %)	3	0.55	17	1.03	0.2995	0.055
Pre-eclampsia (N, %)	12	2.18	5	0.3	< 0.0001*	0.17
Eclampsia (N, %)	0	0	1	0.06	1.0000*	0.035
Gestational hypertension (N, %)	5	0.91	7	0.42	0.1884*	0.06
Diabetes mellitus in pregnancy (N, %)	107	19.45	249	15.09	**0.0161**	0.116
Placenta previa (N, %)	3	0.55	14	0.85	0.5862*	0.036
Premature separation of placenta (N, %)	1	0.18	4	0.24	1.0000*	0.013
Preterm delivery (N, %)	62	11.27	56	3.39	** < 0.0001**	0.306
Pregnancy or postpartum infection (N, %)	111	20.18	232	14.06	**0.0006**	0.163
Delayed fetal growth (N, %)	1	0.18	0	0	0.2500*	0.064
C-sec (N, %)	128	23.27	197	11.94	** < 0.0001**	0.301

*P*-values shown in bold indicate statistically significant differences between the two groups.

*ON* optic neuritis, *SMD* standardized mean difference, *C-sec* cesarean section.

*P-values by Fisher’s exact test, other p-values were anlyazed by Chi-squared test.

**Table 4:** Pregnancy complications in the pregnancy-optic neuritis group compared to the pregnancy-control group.

	Pregnancy-ON group (N = 550), N (%)	Pregnancy-control group (N = 1650), N (%)	Crude OR (95% CI)	Adjusted OR1^a^ (95% CI)	Adjusted OR2 (95% CI)
Pre-eclampsia	12 (2.18)	4 (0.24)	**9.178 (2.948, 28.578)**	**9.189 (2.951, 28.619)**	**8.327 (2.911, 23.819)**^b^
Eclampsia	0 (0)	1 (0.06)	0.999 (0.041, 24.613)	0.993 (0.055, 17.796)	0.616 (0.052, 7.271)^c^
Gestational hypertension	5 (0.91)	7 (0.42)	2.153 (0.681, 6.813)	2.154 (0.681, 6.813)	2.190 (0.749, 6.404)^d^
Diabetes mellitus in pregnancy	107 (19.45)	249 (15.09)	**1.359 (1.058, 1.746)**	**1.359 (1.058, 1.746)**	**1.365 (1.062, 1.754)**^e^
Placenta previa	3 (0.55)	13 (0.79)	0.641 (0.183, 2.239)	0.641 (0.183, 2.239)	0.729 (0.231, 2.302)^f^
Premature separation of placenta	1 (0.18)	3 (0.18)	1.000 (0.104, 9.633)	1.000 (0.104, 9.633)	1.254 (0.251, 6.280)^g^
Preterm delivery	62 (11.27)	52 (3.15)	**3.904 (2.664, 5.722)**	**3.917 (2.671, 5.744)**	**3.914 (2.667, 5.742)**^h^
Pregnancy and postpartum infection	111 (20.18)	217 (13.15)	**1.661 (1.291, 2.138)**	**1.671 (1.296, 2.154)**	
Delay of fetal growth	1 (0.18)	0 (0)	9.011 (0.366, 221.84)	8.952 (0.508, 157.657)	**9.867 (1.224, 79.564)**^i^
C-sec	126 (22.91)	197 (11.94)	**2.237 (1.747, 2.865)**	**2.241 (1.749, 2.871)**	

Odds ratios shown in bold indicate numbers that are statistically significant based on the 95% confidence limit.

*ON* optic neuritis, *OR* odds ratio, *CI* confidence interval, *C-sec* cesarean section.

^a^Adjusted for age of pregnancy.

^b^Adjusted for age of pregnancy, and cancer.

^c^Adjusted for age of pregnancy, cancer, diabetes, hypertension, dyslipidemia, thyroid disease, ovarian dysfunction, and endometriosis.

^d^Adjusted for age of pregnancy, and cancer.

^e^Adjusted for age of pregnancy, diabetes, and dyslipidemia.

^f^Adjusted for age of pregnancy, cancer, ovarian dysfunction, and endometriosis.

^g^Adjusted for age of pregnancy, cancer, hypertension, dyslipidemia, ovarian dysfunction, and endometriosis.

^h^Adjusted for age of pregnancy, and ovarian dysfunction.

^i^Adjusted for age of pregnancy, cancer, diabetes, hypertension, dyslipidemia, thyroid disease, ovarian dysfunction, and endometriosis.

## Discussion

We analyzed 2516 Korean female cohorts aged 18 to 50 years with a history of ON (ON group) and 7548 women control matched with age (control group) for comparing pregnancy and delivery rates. Women with ON history had a 0.72- and 0.65-fold decreased chance of having pregnancy and delivery, after adjusting for age and comorbid diseases. We also analyzed 550 Korean women aged 18 to 55 years who had a pregnancy after ON (pregnancy-ON group) and 1650 age-matched controls who had pregnancy (pregnancy-control group). Pregnant women with ON history were more likely to experience pre-eclampsia, diabetes mellitus in pregnancy, preterm delivery, pregnancy or postpartum infection, delay of fetal growth, and C-sec than pregnant women without a history of ON.

The incidence of ON is usually higher in females than males. A population-based study in the United States of America has estimated that the incidence rate of ON was 2.6 for men and 7.5 for women per 100,000^1^. Moreover, the age of onset of ON is generally between 20 and 49 years, which are child-bearing ages^15,16^. Autoimmune diseases such as MS and NMOSD commonly associated with ON also showed higher prevalence in females than in males^17,18^. There have been studies about the effect of MS and NMOSD on pregnancy and vice versa^11–14,19–23^. Contrary, there is a lack of study about the effect of ON on future pregnancy. This is the first study to present the pregnancy rate and outcomes of pregnancy in women with a history of ON using a population-based cohort. Some ON patients found the underlying diseases at the time of ON attack or during the follow-up. In the present study, as we aimed to display outcomes of pregnancy in subjects who experienced first ON attack without any previous underlying disease that could accompany ON, we made up cohorts consisting of females with ON history without a previous diagnosis of diseases such as MS, NMOSD, ATM, or rheumatic diseases frequently accompanying ON.

In our cohort, the odds of pregnancy was significantly lower in the ON group than in the control group. After adjusting for age and comorbidities, the adjusted odds of pregnancy in the ON group was 71.6% lower than that in the control group. Several factors might have affected the pregnancy rate in women with a history of ON. First, the medication for underlying disease or ON might have affected the fertility or tendency to avoid pregnancy. Short-term use of intravenous corticosteroid treatment is the mainstay of a single episode of ON. However, multiple relapsing conditions and the occurrence of comorbid autoimmune diseases may require long-term maintenance immunosuppressive therapy. Prolonged use of steroids may disrupt the menstrual cycle and cause difficulty in conceiving^24^. Medications such as methotrexate and mycophenolate mofetil can cause serious fetal abnormality. Thus contraception is recommended. Cyclophosphamide treatment may induce ovarian failure and cause reduced fertility^25^. Secondly, regarding a relatively low prevalence of other comorbid autoimmune diseases in our ON cohort, autoimmunity underlying ON itself might have affected fertility. Several autoimmune diseases, such as autoimmune thyroid disease^26,27^ and antiphosphlipid syndrome^28,29^, are known to potentially impair female fertility. However, the causative relationship between these disease and impaired fertility is still debated. It also has been reported that approximately 15–30% of couples who are unable to conceive are diagnosed with unexplained infertility^30^. Immune dysregulation with increased production of autoantibodies is a potential etiologic candidate for this group of patients^31^.

In the present study, the adjusted odds of delivery in the ON group was 64.7% lower than that in the control group. On the contrary, the adjusted odds ratio of abortion was not significant in the ON group compared to the control. Previous studies have shown that the rate of spontaneous abortions in women with MS seems to be comparable to that in the general population^32,33^ However, several studies have shown that women with NMOSD have an increased risk of miscarriage^14,34,35^. One of the possible causes of miscarriage in women with NMOSD is damage to the placenta by circulating AQP4 antibodies. AQP4 antibodies can pass the blood-placenta barrier and cause inflammatory changes, and placental necrosis^36^.

In this study, pregnant women with ON cohort showed 8.3-, 1.37-, 3.91-, 1.67-, 9.86-, and 2.24-fold of increased risk for having pre-eclampsia, diabetes in pregnancy, preterm deliver, pregnancy and postpartum infection, delay of fetal growth, and C-sec, respectively, after adjusting comorbid diseases than the age-matched pregnancy-control group. In previous studies using the United States administrative claims data, women with MS had higher risks of having a preterm delivery and infection in pregnancy, but not significantly higher risk of pre-eclampsia than women without MS^11,13^. Another study using hospital discharge data has found that rates of urinary tract infection, C-sec, and induction of labor, but not antepartum and peripartum morbidities, are slightly increased in women with MS^21^. Women with NMOSD have an increased risk of pre-eclampsia in several studies^14,34,35^. Women with systemic lupus erythematosus, which has peak incidence during the 20s, are also frequently associated with pre-eclampsia, pregnancy loss, delay of fetal growth, preterm birth, and C-sec^37^. However, in the pregnancy-ON cohort of this study, proportions of NMOSD, MS, and other autoimmune diseases were relatively very low (each less than 0.4%). Considering such low proportions of these diseases and relatively large differences in rates of several complications such as pre-eclampsia, preterm delivery, and infection between the two groups, different complication rates cannot be fully explained by the effects of these accompanying diseases. Other factors such as medical comorbidities might also affect pregnancy complications. In an effort to compensate for this, we evaluated the odds ratio for diverse possible underlying diseases. Any disease that showed a significant association with the outcomes of interest was adjusted for OR2. NMOSD and MS were also adjusted in the analysis of multiple adverse outcomes including pre-eclampsia and delay of fetal growth. Even after adjusting for outcome-related variables, the odds ratios of ON for pregnancy complications remained similar. This suggests that ON itself, whether the key factor is underlying autoimmunity or other pathomechanisms accompanying ON, may impact pregnancy outcomes and lead to serious adverse events related to pregnancy.

This retrospective cohort analysis using the claims database has some limitations. We had to make operational definitions for pregnancy, delivery, and abortion, which was an inevitable limitation of a study using a health claim database. However, this study was based on the national health insurance health claim database encompassing the whole South Korean population, enabling us to make a cohort of women with ON history of childbearing age and matched controls. In addition, when matching controls with age, we tried to find possible confoundings based on a literature review, although, unknown and unmeasured variables might result in residual confoundings. Furthermore, as this study is based on insurance claims data, there may be differences between the actual disease incidence rate and the insurance claim rate. Moreover, most of our cohort were Koreans. There might be limits when applying the results of this study to other races.

In summary, this population-based retrospective cohort study using a health claim database of South Korea found that women with ON history had significantly decreased odds of having pregnancy and delivery compared to age-matched controls. Pregnant women with ON history showed a significantly increased risk of having pregnancy complications such as pre-eclampsia, diabetes in pregnancy, preterm delivery, pregnancy and postpartum infection, and delay of fetal growth than age-matched controls.

## Supplementary Information

Supplementary Tables.

## Data Availability

The data that support the findings of this study are available from Health Insurance Review and Assesment (HIRA) of South Korea. However, HIRA’s audited approval is mandatory to access to theses data, so are not publicly available, but are available from corresponding author upon reasonable request.

## Abbreviations

AQP4: Aquaporin 4
ATM: Acute transverse myelitis
C-sec: Cesarean section
HIRA: Health Insurance Review and Assessment
MOGAD: Myelin oligodendrocyte glycoprotein antibody-associated disease
MS: Multiple sclerosis
NMOSD: Neuromyelits optica spectrum disorders
OR: Odds ratio
RDR: Rare Diseases Registry
