# Supplementary material for: Pregnancy prevalence and outcomes after optic neuritis in South Korea
Source: Sci Rep. 2023 Jul 27;13:12167. doi: 10.1038/s41598-023-38851-x (PMC10374557; doi:10.1038/s41598-023-38851-x)
Supplement: Supplementary file 1 — Supplementary Tables. [file 41598_2023_38851_MOESM1_ESM.docx]

Supplemental Table 1. Definitions of Pregnancy, Abortion, Delivery, and Pregnancy Complications

| Definition |  | KCD-7 codes |
| --- | --- | --- |
| Pregnancy | Abortion  Pregnancy complications  Delivery complications  Delivery  Health services related to pregnancy | O00, O01, O02, O03, O04, O05, O06, O07, O08,  O20, O21, O22, O23, O24, O25, O26, O28, O29,  O30, O31, O32, O33, O34, O35, O36, O40 O41, O42, O43, O44, O45, O46, O47, O48,  O60, O61, O62, O63, O64, O65, O66, O67, O68, O69, O70, O71, O82, O73, O74, O75,  O80, O81, O82, O83, O84,  Z31, Z33, Z34, Z35, Z36, Z37, Z38 |
| Abortion |  | O00, O01, O02, O03, O04, O05, O06, O07, O08 |
| Delivery |  | O80, O81, O82, O83, O84 |
| Pregnancy complications | |  |
|  | Pre-eclampsia | O14 |
|  | Eclampsia | O15 |
|  | Gestational hypertension | O13 |
|  | DM in pregnancy | O24 |
|  | Placenta previa | O44 |
|  | Premature separation of placenta | O45 |
|  | Preterm delivery | O60 |
|  | Infection during pregnancy and postpartum | O23, O85, O86, N10, N12, N159 |
|  | Delay of fetal growth | P05, P07 |
|  | C-sec | O82 |

KCD = Korean Standard Classification of Disease; DM = diabetes mellitus; C-sec = Cesarian section.

Supplemental Table 2. Definition of Comorbid Diseases

| Disease | KCD-7 codes |
| --- | --- |
| Multiple sclerosis | V022, G35 |
| Neuromyelitis optica spectrum disorder | G36.0 |
| Acute transverse myelitis | G37.3 |
| Systemic lupus erythematosus | V136, M32 |
| Bechet or Sjorgen syndrome | V139, M35.2, M35.0 |
| Sarcoidosis | V111, D86.X |
| Crohn’s disease | V130, K50 |
| Ulcerative colitis | V131, K51 |
| Acute disseminated encephalomyelitis | G36.8, G36.9, G04.0, G04.8, G04.9 |
| Autoimmune thyroiditis | E06.3, E06.9 |
| Acute rheumatic fever | I00-I02 |
| Rheumatic arthritis | M05, M06 |
| Cancer | C code, V193 |
| Diabetes mellitus | E11, E12, E13, E14 |
| Hypertension | I10, I11, I12, I13, I15 |
| Dyslipidemia | E78 |
| Alcohol addiction | F10 |
| Thyroid disease | E03, E05, E06, E07 |
| Obesity | E66 |
| Ovarian dysfunction | E28 |
| Endometriosis | N80 |

KCD = Korean standard Classification of Disease.

Any diseases with V- code were defined as those having the disease if they had more than one visit under the V-code. Rheumatic arthritis was defined as having a code of M05 or M06 with at least one claim and a prescription for biologics (adalimumab, etanercept, infliximab, rituximab) or any disease-modifying antirheumatic drugs (azathioprine, bucillamine, cyclosporin, D-penicillamine, hydroxychloroquine, leflunomide, methotrexate, minocycline, mizoribine, sulfasalazine, and tacrolimus) in the one year. Autoimmune thyroiditis was defined as having more than one claim with autoimmune thyroiditis code E06.3 or E06.9 and prescription of thyroid hormone supplement. Diabetes was defined as having a code of E11, E12, E13, or E14 and a prescription of hypoglycemic agents. Hypertension was defined as having code of I10, I11, I12, I13, or I15 and prescriptions of antihypertensive medication. Dyslipidemia was defined as having code E78 and a prescription of lipid-lowering medication. Other diseases not having a V- code were defined as having the disease if the subject visited more than two visits under each disease code.

Supplemental Table 3. Crude Odds Ratios of Pregnancy, Delivery, and Abortion According to Comorbid Disease Status

|  | **Pregnancy** | | | **Delivery** | | | **Abortion** | | |
| --- | --- | --- | --- | --- | --- | --- | --- | --- | --- |
|  | **Crude OR** | **95% CI** | ***P*-value** | **Crude OR** | **95% CI** | ***P*-value** | **Crude OR** | **95% CI** | ***P*-value** |
| Age at baseline | 0.897 | 0.892, 0.903 | **<0.0001** | 0.898 | 0.891, 0.905 | **<0.0001** | 0.931 | 0.920, 0.942 | **<0.0001** |
| Multiple sclerosis | 0.647 | 0.300, 1.396 | 0.2670* | 0.652 | 0.270, 1.576 | 0.3418* | 0.593 | 0.116, 3.033 | 0.5300* |
| Neuromyelitis optica | 0.567 | 0.264, 1.216 | 0.1448* | 0.574 | 0.239, 1.379 | 0.2146* | 0.523 | 0.102, 2.675 | 0.4365* |
| Acute transverse myelitis | 0.975 | 0.239, 3.983 | 0.9720* | 0.771 | 0.134, 4.425 | 0.7705* | 0.851 | 0.046, 15.769 | 0.9140* |
| Systemic lupus erythematosus | 0.488 | 0.062, 3.817 | 0.4944 | 0.301 | 0.016, 5.814 | 0.4268* | 1.072 | 0.056, 20.694 | 0.9632* |
| Bechet or Sjorgen syndrome | 0.542 | 0.126, 2.339 | 0.4119 | 0.772 | 0.179, 3.331 | 0.7286 | 1.304 | 0.174, 9.757 | 0.7963 |
| Sarcoidosis | 0.323 | 0.015, 6.961 | 0.4707* | 0.463 | 0.022, 9.874 | 0.6222* | 1.648 | 0.077, 35.162 | 0.7489* |
| Crohn;s disease | 4.888 | 0.306, 78.185 | 0.2619 | 6.954 | 0.435, 111.249 | 0.1704 | 4.928 | 0.119, 204.036 | 0.4012* |
| Ulcerative colitis | 1.964 | 0.381, 10.115 | 0.4197 | 1.158 | 0.139, 9.630 | 0.8918 | 1.648 | 0.077, 35.162 | 0.7489* |
| Acute disseminated encephalomyelitis | 1.439 | 0.530, 3.905 | 0.4750 | 1.549 | 0.524, 4.581 | 0.4292 | 0.549 | 0.031, 9.630 | 0.6813* |
| Autoimmune thyroiditis | 1.63 | 0.740, 3.590 | 0.2255* | 0.796 | 0.259, 2.451 | 0.6911* | 2.848 | 0.922, 8.794 | 0.0688* |
| Acute rheumatic fever | 1.222 | 0.136, 10.935 | 0.8580 | 1.745 | 0.195, 15.584 | 0.6181 | 2.235 | 0.093, 53.691 | 0.6201* |
| Rheumatic arthritis | 0.442 | 0.167, 1.173 | 0.1013* | 0.197 | 0.038, 1.017 | 0.0524* | 1.199 | 0.332, 4.336 | 0.7820* |
| Cancer | 0.712 | 0.523, 0.968 | **0.0301** | 0.664 | 0.462, 0.954 | **0.0266** | 0.887 | 0.505, 1.557 | 0.6766 |
| Diabetes mellitus | 0.525 | 0.396, 0.696 | **<0.0001** | 0.503 | 0.361, 0.700 | **<0.0001** | 0.81 | 0.501, 1.310 | 0.3902 |
| Hypertension | 0.276 | 0.204, 0.372 | **<0.0001** | 0.315 | 0.227, 0.439 | **<0.0001** | 0.286 | 0.152, 0.537 | **0.0001** |
| Dyslipidemia | 0.506 | 0.429, 0.597 | **<0.0001** | 0.471 | 0.387, 0.572 | **<0.0001** | 0.637 | 0.466, 0.870 | **0.0046** |
| Alcohol addiction | 0.430 | 0.132, 1.402 | 0.1616 | 0.614 | 0.189, 1.999 | 0.4180 | 0.328 | 0.019, 5.558 | 0.4403* |
| Thyroid disease | 1.143 | 0.981, 1.333 | 0.0871 | 1.117 | 0.939, 1.330 | 0.2115 | 1.593 | 1.221, 2.077 | **0.0006** |
| Obesity | 0.444 | 0.104, 1.888 | 0.2713 | 0.302 | 0.041, 2.236 | 0.2413 | 1.079 | 0.146, 7.988 | 0.9410 |
| Ovarian dysfunction | 2.856 | 1.947, 4.190 | **<0.0001** | 2.375 | 1.553, 3.633 | **<0.0001** | 4.764 | 2.851, 7.962 | **<0.0001** |
| Endometriosis | 1.016 | 0.710, 1.453 | 0.9311 | 0.956 | 0.632, 1.446 | 0.8306 | 1.212 | 0.637, 2.306 | 0.5571 |

Reference: without each comorbid disease.

*P*-values marked with bold indicate statistically significant differences between the two groups.

OR = Odds ratio; CI = Confidence interval.

Supplemental Table 4. Crude Odds Ratios of Pregnancy Complications According to Comorbid Status

|  | **Pre-eclampsia** | **Eclampsia** | **Gestational hypertension** | **DM in pregnancy** | **Placenta previa** | **Premature separation of placenta** | **Preterm delivery** | **Pregnancy & postpartum infection** | **Delay of fetal growth** | **C-sec** |
| --- | --- | --- | --- | --- | --- | --- | --- | --- | --- | --- |
| age of pregnancy | 0.951 (0.858, 1.054) | 1.156 (0.907, 1.472) | 1.017 (0.919, 1.124) | 1.001 (0.980, 1.022) | 0.975 (0.886, 1.072) | 1.022 (0.861, 1.213) | 0.962 (0.925, 0.999) | 0.942 (0.919, 0.965) | 0.749 (0.446, 1.260) | 0.977 (0.954, 0.999) |
| Multiple sclerosis | **44.712 (0.479, 999.999)** | 178.934 (3.853, 999,999) | **57.605 (0.594, 999,999)** | 1.730 (0.019, 159.457) | **42.259 (0.454, 999.999)** | **165.634 (1.665, 999.999)** | 6.179 (0.068, 562.117) | 17.208 (0.184, 999.999) | **178.934 (3.853, 999.999)** | 1.925 (0.021, 177.612) |
| Neuromyelitis optica | **26.436 (0.621, 999.999)** | **292.917 (5.108, 999.999)** | **34.949 (0.813, 999,999)** | 1.031 (0.025, 42.696) | **24.908 (0.586, 999.999)** | **97.567 (2.115, 999.999)** | 3.632 (0.087, 150.864) | 5.678 (0.354, 91.010) | **292.917 (5.108, 999.999)** | 5.790 (0.361, 92.796) |
| Bechet or Sjorgen syndrome | **44.712 (0.479, 999.999)** | **478.934 (3.853, 999.999)** | **57.605 (0.594, 999,999)** | 1.730 (0.019, 159.457) | **42.259 (0.454, 999.999)** | **165.634 (1.665, 999.999)** | 6.179 (0.068, 562.117) | 1.899 (0.021, 175.073) | **478.934 (3.853, 999.999)** | 1.925 (0.021, 177.612) |
| Autoimmune thyroiditis | **140.804 (8.422, 999.999)** | **292.917 (5.108, 999.999)** | **190.341 (1.213, 999.999)** | 1.031 (0.025, 42.696) | **24.908 (0.586, 999.999)** | **97.567 (2.115, 999.999)** | 3.632 (0.087, 150.864) | 5.678 (0.354, 91.010) | **292.917 (5.108, 999.999)** | 5.790 (0.361, 92.796) |
| Rheumatic arthritis | **44.712 (0.479, 999.999)** | **478.934 (3.853, 999.999)** | **57.605 (0.594, 999.999)** | 1.730 (0.019, 159.457) | **42.259 (0.454, 999.999)** | **165.634 (1.665, 999.999)** | 6.179 (0.068, 562.117) | 1.899 (0.021, 175.073) | **478.934 (3.853, 999.999)** | 1.925 (0.021, 177.612) |
| Cancer | **12.004 (0.485, 297.038)** | **132.972 (3.808, 999.999)** | **15.868 (0.634, 397.442)** | 1.296 (0.144, 11.628) | **11.313 (0.458, 279.354)** | **44.281 (1.628, 999.999)** | 1.651 (0.069, 39.631) | 0.516 (0.022, 12.298) | **132.972 (3.808, 999.999)** | 0.523 (0.022, 12.477) |
| DM | 2.293 (0.128, 41.108) | **25.389 (0.969, 665.018)** | 3.032 (0.167, 55.077) | **2.491 (1.118, 5.552)** | 2.160 (0.121, 38.661) | **8.449 (0.424, 168.506)** | 0.315 (0.018, 5.463) | 1.240 (0.468, 3.285) | **25.389 (0.969, 665.018)** | 0.440 (0.104, 1.864) |
| Hypertension | 5.268 (0.267, 103.859) | **58.351 (2.049, 999.999)** | 6.965 (0.349, 139.080) | 1.037 (0.226, 4.750) | 4.965 (0.252, 97.662) | **19.424 (0.889, 424.214)** | 0.724 (0.038, 13.828) | 0.225 (0.012, 4.294) | **58.351 (2.049, 999.999)** | 0.523 (0.067, 4.065) |
| Dyslipidemia | 0.835 (0.049, 14.306) | **9.252 (0.368, 232.679)** | 2.561 (0.326, 20.096) | **1.768 (1.038, 3.009)** | 0.787 (0.046, 13.450) | **9.427 (0.969, 91.689)** | 1.017 (0.365, 2.835) | 0.857 (0.436, 1.685) | **9.252 (0.368, .232.679)** | 0.769 (0.379, 1.557) |
| Alcohol addiction | **26.436 (0.621, 999.999)** | **292.917 (5.108, 999.999)** | **34.949 (0813, 999.999)** | 1.031 (0.025, 42.696) | **24.908 (0.586, 999.999)** | **97.567 (2.115, 999.999)** | 3.632 (0.087, 150.864) | 5.678 (0.354, 91.010) | **292.917 (5.108, 999.999)** | 1.148 (0.028, 47.559) |
| Thyroid disease | 1.688 (0.220, 12.928) | **8.345 (0.333, 209.385)** | 2.306 (0.294, 18.068) | 1.132 (0.640, 2.001) | 1.581 (0.207, 12.066) | 2.778 (0.146, 52.838) | 1.165 (0.463, 2.937) | 0.946 (0.507, 1.763) | **8.345 (0.333, 209.385)** | 0.773 (0.395, 1.512) |
| Obesity | **14.678 (0.540, 399.084)** | **162.653 (4.280, 999. 999)** | **19.428 (0.707, 533.592)** | 2.223 (0.261, 18.913) | **13.827 (0.509, 375.391)** | **54.167 (1.818, 999.999)** | 2.018 (0.076, 53.304) | 0.629 (0.024, 16.557) | **162.653 (4.280, 999.999)** | 2.477 (0.291, 21.077) |
| Ovarian dysfunction | 1.783 (0.101, 31.476) | **19.757 (0.765, 510.036)** | 2.359 (0.132, 42.164) | 0.644 (0.227, 1.832) | **3.838 (0.495, 29.728)** | **20.583 (2.089, 202.769)** | 3.041 (1.160, 7.974) | 0.916 (0.354, 2.373) | **19.757 (0.765, 510.036)** | 0.520 (0.159, 1.706) |
| Endometriosis | 3.196 (0.173, 59.066) | **35.432 (1.319, 951.848)** | 4.230 (0.226, 79.090) | 1.298 (0.432, 3.907) | **16.037 (3.415, 75.303)** | **11.796 (0.576, 241.705)** | 0.440 (0.025, 7.850) | 0.298 (0.040, 2.228) | **35.432 (1.319, 951.848)** | 1.018 (0.297, 3.495) |

*P*-value < 0.2 are displayed in bold text.

DM = diabetes mellitus; C-sec = Cesarean section.
